# Direct visualization and correlation of liver stereotactic body radiation therapy treatment delivery accuracy with interfractional motion

**DOI:** 10.1002/acm2.13333

**Published:** 2021-07-09

**Authors:** S. Kuznetsova, R. Sinha, K. Thind, N. Ploquin

**Affiliations:** ^1^ Department of Physics and Astronomy University of Calgary Calgary AB Canada; ^2^ Department of Oncology University of Calgary Calgary AB Canada

**Keywords:** interfractional motion, liver, Primovist MRI, stereotactic body radiation therapy (SBRT), treatment delivery accuracy

## Abstract

This study used the visualization of hypo‐intense regions on liver‐specific MRI to directly quantify stereotactic body radiation therapy (SBRT) spatial delivery accuracy. Additionally, the interfractional motion of the liver region was determined and compared with the MRI‐based evaluation of liver SBRT spatial treatment delivery accuracy. Primovist^®^‐enhanced MRI scans were acquired from 17 patients, 8–12 weeks following the completion of liver SBRT treatment. Direct visualization of radiation‐induced focal liver reaction in the form of hypo‐intensity was determined. The auto‐delineation approach was used to localize these regions, and center‐of‐mass (COM) discrepancy was quantified between the MRI hypo‐intensity and the CT‐based treatment plan. To assess the interfractional motion of the liver region, a planning CT was registered to a Cone Beam CT obtained before each treatment fraction. The interfractional motion assessed from this approach was then compared against the localized hypo‐intense MRI regions. The mean ± SD COM discrepancy was 1.4 ± 1.3 mm in the left‐right direction, 2.6 ± 1.8 mm in an anteroposterior direction, and 1.9 ± 2.6 mm in the craniocaudal direction. A high correlation was observed between interfractional motion of visualized hypo‐intensity and interfractional motion of planning treatment volume (PTV); the quantified Pearson correlation coefficient was 0.96. The lack of correlation was observed between Primovist^®^ MRI‐based spatial accuracy and interfractional motion of the liver, where Pearson correlation coefficients ranged from −0.01 to −0.26. The highest random and systematic errors quantified from interfractional motion were in the craniocaudal direction. This work demonstrates a novel framework for the direct evaluation of liver SBRT spatial delivery accuracy.

## 1 INTRODUCTION

Stereotactic body radiation therapy (SBRT) is a treatment technique characterized by high radiation doses delivered over a shorter period with a steep dose gradient.^1^ SBRT has high local control rates for liver malignancies,^1^, ^2^, ^3^, ^4^, ^5^, ^6^, ^7^ leading to an effective and non‐invasive treatment alternative for patients with liver lesions. High spatial delivery accuracy is essential in SBRT treatment.^8^ The presence of intra‐ and interfractional motion due to liver deformation and liver motion may lead to the dose delivered to differ from the intended treatment plan.^9^

Gadolinium‐ethoxybenzyl‐diethylenetriamine pentaacetic acid (Gd‐EOB‐DTPA, Primovist^®^) is a magnetic resonance imaging (MRI) contrast agent, which is taken up by hepatocyte cells within the liver parenchyma. This results in a hepatobiliary contrast phase along with dynamic imaging capabilities.^10^ Primovist^®^‐enhanced MRI can be utilized to characterize liver lesions, and the hepatobiliary contrast phase can help to visualize and assess hepatic function.^11^ Because of this property, the Primovist^®^ contrast is used to assess post‐SBRT liver intensity changes. The hepatocyte‐specific property of the contrast agent results in a well‐demarcated hypo‐intensity observed on a hepatobiliary imaging phase 1–6 months post‐treatment.^12^ Past studies were able to correlate the intensity signal of liver parenchyma post‐Primovist^®^ administration to liver function.^11^ The study by Okamoto et al. observed signal intensity decrease with the increase of EQD and BED of the irradiated area.^13^ The study by Jung et al. obtained pre‐treatment and post‐treatment Primovist^®^‐based MRI and observed an increase in intensity change, defined as the difference between a pre‐treatment and post‐treatment MRI intensity, with the increase in radiation dose.^14^ The hypo‐intense regions depicted on an MRI are typically referred to as a Focal Liver Reaction (FLR).^10^ A combination of Primovist^®^‐enhanced MRI, along with the dose distribution from the planning CT (pCT) can be used to estimate radiation dose levels associated with the development of the FLR. This is referred to as the threshold dose.^15^ Furthermore, the hypo‐intense regions allow for the assessment of the planned delivery compared with the spatial end‐result of delivery. This quantitative assessment framework based on Primovist^®^‐MRI allows for a direct estimation of spatial radiation dose‐delivery accuracy.

Past studies that used Primovist^®^‐based enhanced MRIs to either determine FLR threshold dose or spatial delivery accuracy used manual contouring of the hypo‐intense regions.^10^, ^11^, ^12^, ^13^, ^16^, ^17^, ^18^, ^19^, ^20^ Manual contouring introduces intra‐ and interobserver variability, especially around the edges of the region with reduced intensity. Furthermore, past studies have used pCT dose distributions to assess the threshold dose associated with the FLR. However, that does not take into account interfractional motion (i.e., day‐to‐day variations in the treatment setup). These studies assume that the dose distribution generated by a treatment planning software stays consistent throughout fractionated treatment delivery. However, studies assessing interfractional variability for liver SBRT have found the mean liver shape changes to be 4.6 mm^21^ and significant differences in the planned and delivered dose distributions.^22^

The three major objectives of this study were as follows:

1. Compute and compare FLR threshold dose with and without interfractional motion.
2. Quantify the spatial delivery accuracy of liver SBRT using Primovist^®^ MRI.
3. Quantify interfractional motion of liver, FLR, and PTV regions and assess the correlation with Primovist^®^ MRI determined spatial delivery accuracy.

## 2 METHODS

### 2.1 Patient selection and liver SBRT treatment

This study retrospectively analyzed a cohort of 17 patients treated with liver SBRT for oligometastases. Diagnostic images were acquired using a Primovist^®^‐MRI. The SBRT treatment planning protocol follows institutional standards, and consists of a 4DCT acquisition (1.37 × 1.37 mm pixel spacing and 3 mm slice thickness) where patients are immobilized using a Vac‐Lok^TM^ bag along with abdominal compression for motion management (CDR Freedom System^TM^). Patients must lay flat and tolerate abdominal compression. Gross tumor volume (GTV) and/or internal target volume (ITV) were delineated on a 4DCT using Eclipse software (Varian Medical Systems, Inc, Palo Alto, CA, USA). An isotropic margin of 5 mm was added to the ITV to define the planning target volume (PTV). The SBRT protocol prescribes a dose of 50 Gy over 5 fractions (*BED_10_* = 100 Gy) for *Child*‐*Pugh*‐*A* liver function, and a 95% isodose line to the PTV. For *Child*‐*Pugh*‐*B* grade liver, the total prescription dose was lowered to 30–35 Gy over 5 fractions (*BED_10_* = 48–59.5 Gy). If the average dose for a healthy liver was exceeded, then the total prescription dose was lowered in 5 Gy increments. Cone beam CTs (CBCT) were acquired for patients at each treatment fraction. An automatic match using bone and liver diaphragm were performed between CBCT and pCT. When adjustment to the automatic match was required, particular attention was given to the liver and adjacent abdomen anatomy in the vicinity of the PTV. If the GTV is visible on a CBCT, then it had to be within the PTV contour. Following the rigid registration, appropriate treatment couch shifts were applied in order to better align a patient to the treatment plan position. The VMAT treatment was delivered using Varian TrueBeam linear accelerators with 6 or 10 MV flattening filter‐free (FFF) co‐planar beams.

### 2.2 Post‐SBRT Primovist^®^‐enhanced MRI acquisition

The Primovist^®^‐enhanced MRI acquisition was performed 8–12 weeks post‐SBRT treatment delivery. The images were acquired with a 1.5T GE Medical Systems Optima MR360 or Siemens Aera scanner with a spoiled 3D Gradient Echo sequence under a breath‐hold condition. The resulting T1‐weighted images had a pixel spacing ranging from 0.7 to 1.2 mm with slice thickness between 2.5 and 3.5 mm. Imaging at the hepatobiliary phase was performed 15–20 min after the intravenous injection of the Primovist^®^ contrast agent. The hepatobiliary‐phase MRI scans were used for FLR visualization and delineation.

### 2.3 FLR threshold dose quantification

The FLR threshold dose was quantified using a pCT and post‐SBRT MRI. Deformable image registration (DIR) in Velocity AI (Varian Medical Systems, Inc, Palo Alto, CA, USA) was used to register the two image sets. The details regarding the image registration implementation and performance assessment have been previously described.^23^, ^24^ Following image registration, in‐house software was used for localization of the hypo‐intensity. The custom program was developed in MatLab R2016a. This program finds appropriate thresholding from the correlation of intensity and dose information from the treatment plan.^25^

In order to quantify the threshold dose associated with the FLR, the dice similarity coefficient (DSC) conformality metric was used between the localized FLR structure (*V_FLR_*) and various isodose lines (*V_Dose_*). The dose structure with the highest DSC was defined as the FLR threshold dose (*D_thr_*). Using the DSC formulism, the D_thr_ for the planned dose distribution was determined (D_P_thr_). It is assumed that a dose threshold exists below which the Primovist^®^ contrast uptake is consistent.

**FIGURE 1:**
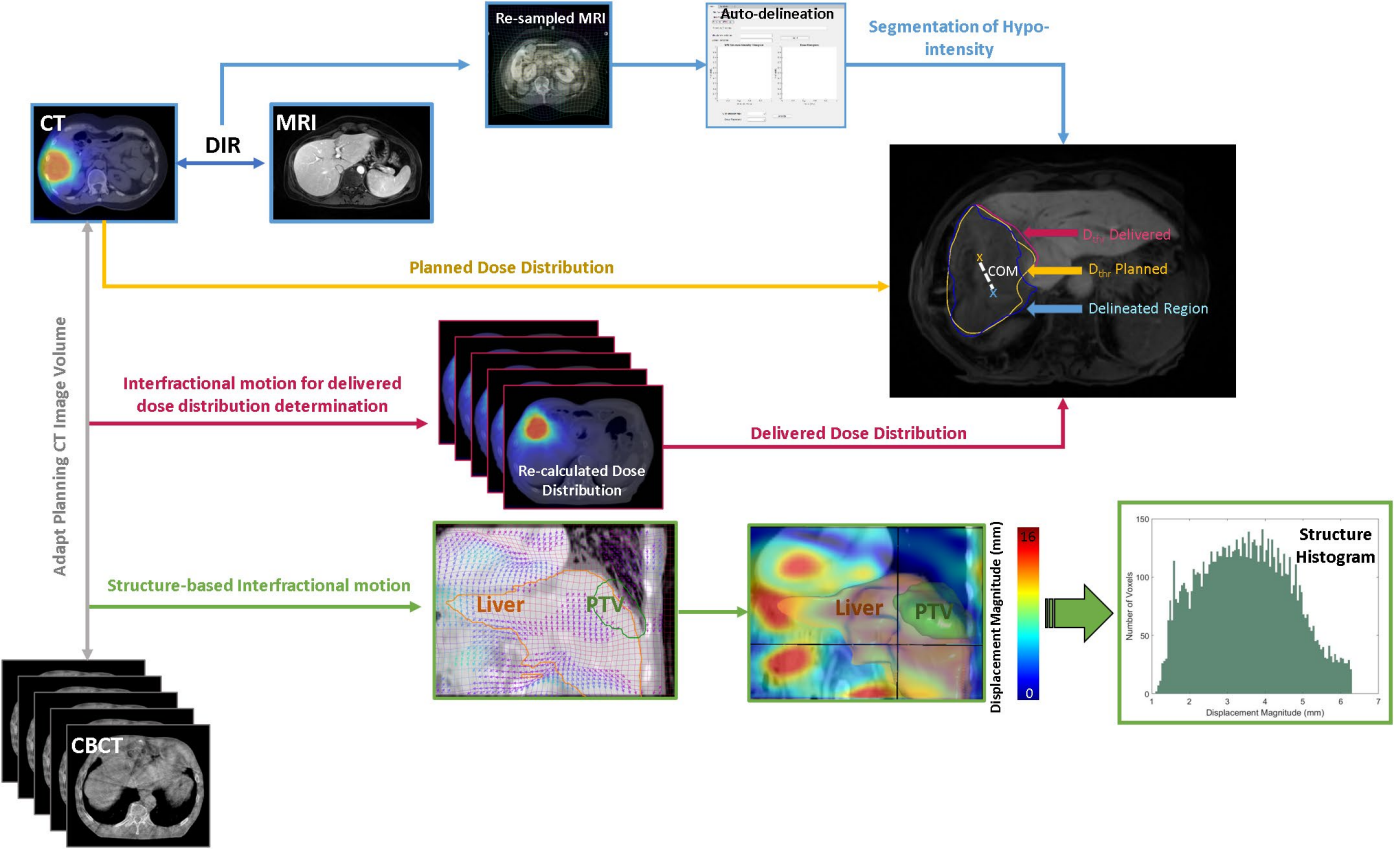
Workflow describing the creation of the delineated region of observed hypo‐intensity on Primovist^®^‐enhanced MRI, as well as creation of planned and delivered threshold dose structures, and quantification of interfractional motion for liver, FLR, and PTV structures. The region of hypo‐intensity (i.e., end‐result of radiation delivery) was visualized on post‐SBRT MRI, which was registered to pCT. The radiation delivery accuracy was quantified by calculating the COM discrepancy between the hypo‐intensity and the D_P_thr_ structure. CBCTs were used to quantify interfractional motion for the purposes of generating delivered dose distribution. CBCTs were also used to quantify interfractional motion in terms of voxel displacement, where displacement histograms for the liver, FLR, and PTV structures were built from 0th to 95th percentile

CBCTs were used to generate delivered dose distributions to assess the impact of interfractional motion on the FLR D_thr_. The delivered dose distribution was generated using the dose accumulation approach.^22^ The dose accumulation technique is based on CBCT images obtained at each treatment delivery and pCT.^26^, ^27^, ^28^, ^29^ In our study, we performed DIR between each CBCT (obtained for each fraction) and pCT. Using DIR, pCT volume was deformed to the daily CBCT, thus creating a new image volume while maintaining voxel values from the original pCT.^30^ The treatment plan was applied to the deformed image volume, and the radiation dose was re‐calculated within the Eclipse treatment planning system. The radiation dose was recalculated for each fraction of the liver SBRT treatment. The re‐calculated dose distributions from all fractions were summed to create total delivered dose distribution (Figure 1). The threshold dose associated with the delivered dose distribution (D_D_thr_), which considers the impact of interfractional motion, was determined using DSC.

The Wilcoxon‐signed rank test was performed using SPSS Statistics V23 software (IBM Corporation, USA) to assess whether interfractional motion significantly affects the quantification of the threshold dose associated with the FLR. To compare our results with previously published studies, the D_D_thr_ and D_P_thr_ were converted to biologically equivalent dose (BED) using a linear quadric formalism with $\frac{\alpha }{\beta }=2,\;\frac{\alpha }{\beta }=3,\;\mathrm{and}\;\frac{\alpha }{\beta }=10$, as well as converted to an equivalent dose (EQD_2_) of 2 Gy per fraction.

### 2.4 Spatial treatment delivery accuracy quantification

The geometric center‐of‐mass (COM) was calculated on a localized FLR region, as well as on the corresponding threshold dose (D_P_thr_) structure. Subsequently, the magnitudes of COM discrepancy between the FLR and the threshold dose structure (D_p_thr_) was calculated in three spatial directions: left‐right, anteroposterior, and craniocaudal. This study also calculated the magnitude of the 3D vector between COMs. One of the outputs of the framework, besides the localization of hypo‐intensity, includes an estimation of the COM discrepancy error associated with the localization.^31^

### 2.5 Interfractional motion quantification

The patient setup variations for each treatment fraction were determined by registering CBCT to pCT (Figure 1). The image registration was conducted within Velocity AI software, which uses a B‐spline algorithm. The subsequent image registration was assessed qualitatively, with an emphasis on the liver boundary. The synthetic CT was generated following the image registration with propagated structures from the original CT. The propagated liver contour was qualitatively assessed on the synthetic CT and its conformity to the liver structure. The magnitude of the displacement of each voxel of the pCT image volume in three directions (left‐right, anteroposterior, and craniocaudal) was then quantified. The voxel displacement information was exported for each fraction as an absolute displacement vector field (DVF). The displacement histograms were built for the liver, FLR, and PTV structures with a custom program written in MatLab R2016a. Quantifying interfractional motion based on an entire liver organ motion and on the motion of FLR and PTV regions was motivated by the presence of motion variability throughout the liver.^32^ Furthermore, immobilization is typically conducted with attentiveness to reducing motion of the treated region, which can further result in the motion variability throughout the liver. The mean displacement was calculated for each structure from the DVF information. The extracted information was used to quantify random and systematic errors.^33^

The interfractional motion was quantified for each patient and visualized in a 3D space using relative anatomical coordinates. The 2D plane was generated to fit the data using SigmaPlot 14.0 software (©Systat Software Inc., Germany) to help better visualize the data trends. The visualization of interfractional motion was compared against the spatial treatment delivery accuracy as determined using Primovist^®^ MRI. Pearson correlation coefficients were quantified for Primovist^®^ MRI‐based spatial accuracy and interfractional motion. Additionally, Pearson correlation coefficients were quantified for the interfractional motion of PTV and FLR regions.

## 3 RESULTS

### 3.1 Patient characteristics and clinical outcomes

Seventeen patients (20 lesions) underwent liver SBRT with a follow‐up Primovist^®^‐enhanced MRI scan acquisition (Fall 2017 to Summer 2019). Patient characteristics and primary disease sites are summarized in Table 1. The median time between MRI acquisition and the last treatment fraction was 8 weeks. Most patients (12/17) received a total prescription dose of 45–50 Gy.

**TABLE 1:** Patient characteristics

Age (y)	
Median (range)	62(41–86)
Sex	
Male/Female	9/8
ITV Diameter (cm)	
Median (range)	4.6 (1.9–8.8)
Child‐Pugh Score *n(%)*	
No cirrhosis	11(65)
Child‐Pugh A	5(29)
Child‐Pugh B	1(6)
Primary Disease Site *n(%)*	
Liver (HCC)	5(29.4)
Colon	3(17.6)
Lung	2(11.8)
Breast	2(11.8)
Sigmoid	2(11.8)
Other	3(17.6)

Abbreviations: HCC, Hepatocellular Carcinoma; ITV, Internal Target Volume.

Age recorded at the time of planning CT acquisition. ITV size was measured in terms of equivalent sphere diameter for 20 treated lesions.

All the follow‐up (FU) times were measured from pCT acquisition to last follow‐up or death. The median FU was 14.2 months. Local control was achieved in 16/20 (80%) lesions at median FU, and the median time to local recurrence was 317 days. There were 12 /17 (71%) cases with outfield progression: six within the liver (but outside RT field), three lung, one brain, one bone, and one para‐aortic node. The median time to outfield progression was 195 days.

### 3.2 FLR threshold dose

Dose accumulation was performed on 15 patients. Two patients were excluded due to either the absence of CBCTs or the presence of prominent artifacts. The quantified median (range) DSC for the determination of the threshold dose was 0.88 (0.61–0.92) and 0.86 (0.59–0.91) for the planned and delivered dose distributions, respectively. The threshold dose results for the planned and delivered dose distribution are summarized in Table 2 under the row titled “Presented Study.” There was no significant difference observed between D_P_thr_ and D_D_thr_ (*p* = 0.2). The FLR threshold dose inclined to fall closer to the 50% normalized dose range (median D_D_thr_ =52% and D_P_thr_ =56%). The median (range) deviation between D_P_thr_ and D_D_thr_ was 0.5 (0–5) Gy; 11/17 patients had dose deviations of 2 Gy or less between two dose distributions. The calculated medians for D_P_thr_ and D_D_thr_ were the same.

**TABLE 2:** Summary of selected studies that utilized Gd‐EOB‐DTPA ‐based MRI to assess the threshold dose of hypo‐intensity

	Number of patients	Prescription dose and delivery	MRI acquisition post RT (weeks)	Delineation of hypo‐intensity	Image registration (y/n)	Dose distribution	Threshold dose of hypo‐intensity (Gy)
Presented Study	17	Median: 50 Gy/5 fx Linac	8–12	Auto‐delineation	y; DIR	Planned dose & Delivered dose	Median: Absolute Dose:25 BED_α/β=2_=88 BED_α/β=3_=67 BED_α/β=10_ =38 EQD_2,α/β=2_=44
Boda‐Heggemann et al. 2019	n_Linac_=16 n_CyberKnife_=14	Median: 60 Gy/5fx Linac; 40 Gy/3‐5 fx CyberKnife	9–12	Manual	y; Rigid or DIR	Planned dose	Median: BED_α/β=3, Linac_=71.1 BED_α/β=3, Cyberknife_=134.1
Boda‐Heggemann et al. 2018	22	Median: 40 Gy/3‐5 fx CyberKnife	6–17	Qualitative isodose matching	y; Rigid or DIR	Planned dose	Median: EQD_2,α/β=2_ =90.6
Fukugawa et al. 2017	13	45 Gy/15 fx Linac	~4–40	manual	y	Planned dose	Median TD=35.2 BED_α/β=3_=62.7^b^
Jung et al. 2016	20 (17 patients analyzed)	45 Gy/3 fx Linac	8–16	‐	y; Rigid	Planned dose	Median TD=19.8 BED_α/β=3_=63.4^b^
Doi et al. 2016	n_cirrhotic_=54 n_non‐cirrhotic_=10	Median_cirrhotic_: 44 Gy/5 fx; Median_non‐cirrhotic_ 55.5 Gy/5.5 fx Linac	12–24	‐	y; Rigid	Planned dose	Median BED_α/β=2, cirrhotic_=57.3 BED_α/β=2, non‐cirrhotic_=114
Boda‐Heggemann et al. 2016	24	26–28 Gy/ 1 fx (n=11) 60 Gy/5 fx (n=13) Linac	8–12	Qualitative isodose matching	y; Rigid or DIR	Planned dose	Median TD=24 EQD_2,α/β=2_=42
Sanuki et al. 2014	n_CP‐A_=37 n_CP‐B_=13 (45 patients analyzed)	CP‐A: 40 Gy/5 fx CP‐B: 35 Gy/5 fx Linac	4–24	manual	y	Planned dose	Median TD_CP‐A_=30.5 BED_α/β=3,CP‐A_=93^b^ TD_CP‐B_= 25.2 BED_α/β=3,CP‐B_=68^b^
Okamoto et al. 2014	11	30–65.4 Gy/10‐35 fx Linac	1‐67^a^	Qualitative isodose matching	‐	Planned dose	BED_α/β=10_=29–35

Abbreviations: BED, Biologically Equivalent Dose; CP, Child‐Pugh; DIR, Deformable Image Registration; EQD_2_, Equivalent Dose of 2 Gy per fraction; fx, fraction; RT, Radiation Therapy; TD, Threshold Dose.

^a^ Delay from the initiation of RT.

^b^ The value was estimated using the threshold dose and fractionation regimen as stated in the manuscript.

### 3.3 Spatial treatment delivery accuracy

Table 3 shows COM discrepancy between the delineated hypo‐intensity and threshold isodose structures (D_P_thr_). The overall median (range) total COM shift was 4.3 mm (0–9.5), 1.4 mm (0–4.1) in the left‐right direction, and 0 mm (0–9) in the craniocaudal direction. The highest observed discrepancy was 2.7 mm (0–5.5) in the anteroposterior direction. The median (range) total COM error associated with the localization of hypo‐intensity was 0.6 mm (0–3.2).

**TABLE 3:** The COM shift between an auto‐delineated hypo‐intensity (FLR) and threshold isodose structure (D_P_thr_)

	Left‐right (mm)	Anteroposterior (mm)	Craniocaudal (mm)	3D direction (mm)
Median (range)	1.4 (0–4.1)	2.7 (0–5.5)	0 (0–9)	4.3 (0–9.5)
Mean±SD	1.4±1.3	2.6 ±1.8	1.9 ±2.6	4.2 ±2.4

Abbreviations: COM, Centre of Mass; SD, Standard Deviation.

### 3.4 Interfractional motion quantification

The interfractional motion was quantified for the entire liver organ, as well as for the FLR and PTV structures. The mean ± SD of averaged absolute mean displacement magnitudes over all treatment fractions for the liver was 2.2 ± 1.4 mm in the left‐right direction, 2.4 ± 0.9 mm in the anteroposterior direction, and 3.3 ± 2 mm in the craniocaudal direction. The mean ± SD of the average displacement magnitude for the FLR region was 2.3 ± 0.9 mm in the left‐right direction, 2.1 ± 0.9 mm in the anteroposterior direction, and 2.7 ± 1.3 in the craniocaudal direction. The mean ± SD of the averaged absolute mean displacement magnitudes for the PTV structure in the same respective directions was 2.2 ± 0.9 mm, 2.1 ± 1 mm, and 2.7 ± 1.1 mm. The summary of random and systematic errors for the three structures is given in Table 4.

**TABLE 4:** Random systematic errors based on the interfractional motion

		Left‐right (mm)	Anteroposterior (mm)	Craniocaudal (mm)
Liver	σ	0.7	0.7	5.3
	Σ	1.4	0.9	2
FLR	σ	1.2	1.0	1.3
	Σ	0.9	0.9	1.3
PTV	σ	1.1	1.1	1.3
	Σ	0.9	1	1.1

Abbreviations:σ, random error;Σ, systematic error.

**FIGURE 2:**
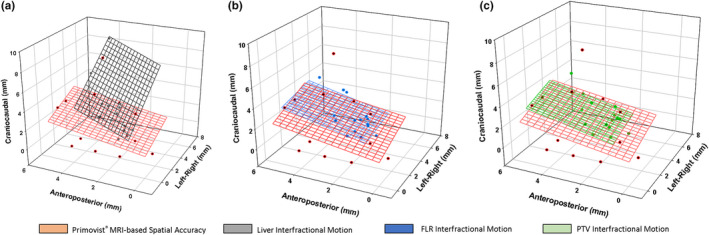
Visualization of spatial delivery accuracy determined using Primovist^®^ MRI along with (a) liver interfractional motion, (b) FLR interfractional motion, and (c) PTV interfractional motion. Each point corresponds to a specific patient. A 2D plane was fitted to delivery accuracy data and interfractional motion data to help better visualize the data trends

The interfractional motion data were visualized in a 3D space using relative anatomical coordinates for Liver, FLR, and PTV regions, along with spatial delivery accuracy as determined by Primovist^®^ MRI (Figure 2).

The Pearson correlation coefficients were quantified between interfractional motion of the three regions (Liver, FLR, and PTV) and Primovist^®^ MRI spatial delivery accuracy for three anatomical directions. Overall, the correlation coefficients were low (0.06–0.26). The highest correlation was observed for anteroposterior direction between Primovist^®^ MRI spatial accuracy and FLR interfractional motion. The lowest correlation was observed in the left‐right direction between FLR and Primovist^®^ MRI.

The correlation was also assessed for the interfractional motion between FLR and PTV regions. The Pearson correlation coefficients were 0.92, 0.98, 0.94, and 0.96 for craniocaudal, anteroposterior, left‐right, and 3D vector magnitude, respectively.

## 4 DISCUSSION

The first objective of this study was to quantify the median threshold dose. This was found to be 25 Gy (BED_α/β = 3_ = 67 Gy, EQD_2_,_α/β = 2_ = 44 Gy) for both planned and delivered dose distributions. Past studies reported a vast range of threshold dose values associated with the observed hypo‐intensity (Table 2) from the Gd‐EOB‐DTPA‐based MRI. The variation in these findings is likely due to different methodologies for radiation delivery, different threshold dose quantification methods, and variability in the timing of the MRI post‐SBRT delivery. The median threshold dose produced by the linear‐quadratic model formulation for BED_α/β = 2_ was 77 to 123 Gy^10^, ^11^, ^12^, ^34^; using a BED_α/β = 3_ formulism, the threshold dose range was reported to be 63–71 Gy.^10^, ^17^, ^34^ Using EQD_2, α/β = 2_ formulism, the threshold dose was reported to be 42 Gy, and 71 Gy using BED_α/β = 3_ formulism for Linac‐based delivery.^16^, ^17^ Our results closely agree with the hypo‐intensity threshold dose reported by past studies that utilized a Linac‐based delivery. Direct comparison with past studies can be challenging due to the differences in fractionation schemes, radiation delivery modalities, and differences in the approaches for determining the threshold dose. In addition, although some studies relied on manual contouring of hypo‐intensity, others used qualitative isodose matching, and few of the studies used either a region‐of‐interest based approach or dose‐intensity histograms. The automated framework presented can help in the unbiased and systematic determination of the threshold dose associated with the FLR, which can be readily adopted into routine clinical practice. This study employed a dose accumulation approach for the assessment of the impact of interfractional motion on the quantified threshold dose. We showed that interfractional motion does not significantly impact the threshold dose associated with the FLR. It was observed that interfractional motion primarily impacts the PTV region of the liver by producing hot‐spot blurring. Due to the median FLR threshold dose falling closer to the 50% normalized dose range, the effects of hot‐spot blurring are negligible in this region. It is important to acknowledge that the effects of interfractional motion on the FLR threshold dose does not translate to the effects that it may have on the PTV coverage. The assessment of the target coverage due to interfractional motion is outside the scope of this study.

The second major objective of this study was the quantification of spatial SBRT treatment delivery accuracy. Automated framework allowed for the FLR localization. The COM was quantified for the FLR structure and compared with the threshold isodose line (D_P_thr_) COM. The median (range) COM shift in the left‐right direction was 1.4 (0–4.1) mm, 2.7 (0–5.5) mm in the anteroposterior direction, and 0 (0–9) mm in the craniocaudal direction. If the COM coordinates were within the same slice, then the calculated COM discrepancy in the CC direction was 0 mm. One of the patients had a CC shift of 9 mm; upon a closer look, the patient's PTV was located in the inferior section of liver segment‐V next to the large bowel. Variations in the bowel filling could have contributed to the large COM shift in the CC direction for this patient case. To our knowledge, only two other studies have attempted to quantify spatial treatment delivery accuracy of liver SBRT using Primovist^®^‐enhanced MRI scans. However, both relied on manual contouring. The median total COM shift error was 4.3 mm. Our results closely align with the results of a study by Boda‐Heggemann et al., where the median COM difference was 4.2 mm.^17^ Furthermore, that same study calculated a median DSC between the hypo‐intensity and the threshold isodose line structures to be 0.80 compared with the higher conformality of 0.86–0.87 in our study. The study by Jung et al. observed a total median COM discrepancy of 6.8 mm.^19^ This study performed manual delineation of the hepatocellular carcinoma and FLR (referred to as parenchymal changes) on MRI scans acquired 2–4 months post‐SBRT completion. Past studies have reported liver volume changes of as much as 20%, 2–6 months after the completion of liver SBRT.^35^, ^36^ Thus, it is important to consider contouring uncertainties and post‐SBRT liver volume changes that may contribute to the COM discrepancy.

In our study, the directional component with the largest median COM shift was observed to be 2.7 mm in the anteroposterior direction. Similarly, a study by Jung et al. has observed the highest median COM discrepancy in the anteroposterior direction (4.1 mm), as well as the study by Boda‐Heggemann et al. observed the highest standard deviation of the mean error to be in the anteroposterior direction. Although our results are consistent with past studies, the exact reason for this behavior remains undetermined. A potential reason for this observation could be the non‐isotropic liver volume change between imaging and radiation delivery, as well as the effects of abdominal compression in some patients.^19^, ^21^

This study quantified the interfractional motion of the liver, FLR, and PTV structures and compared it against Primovist^®^ MRI‐based spatial delivery accuracy. The interfractional motion of PTV and FLR regions was found to be highly correlated. This helps justify the use of visualized hypo‐intensity on Primovist^®^ MRI to estimate the accuracy of liver SBRT spatial delivery. The random and systematic errors were similar between PTV and FLR regions, where differences were less than 0.2 mm. A higher disagreement was found between liver random and systematic errors versus PTV or FLR regions. The differences observed in interfractional motion for the PTV/FLR versus liver structures are expected due to the differences in respiration‐induced motion that can vary by liver segments.^32^ The systematic and random errors for the three structures analyzed were highest in the craniocaudal direction. This contradicts the directly evaluated liver SBRT treatment delivery accuracy results, where the direction of the highest deviation was in the anteroposterior direction. Several past studies have shown a similar influence of interfractional motion. Studies by Kawahara et al. and Lu et al. observed the highest random and systematic errors in the craniocaudal direction ranging from 1.4 to 3.87 mm.^37^, ^38^ A study by Case et al. observed the highest systematic error for patients with abdominal compression in the craniocaudal direction (1.5 mm), and a random error of 1.6 mm in both craniocaudal and anteroposterior directions.^39^ These studies relied on a combination of CBCT imaging and either fiducial markers correspondence, liver motion amplitude change, or whole liver volume centroid correspondence. Study by Eccles et al. used biomechanical DIR (MORFEUS) to assess the displacement of nodes within the liver shape.^21^ This study observed the average interfractional liver shape change to be 2.5 mm in the left‐right direction, 3.6 mm in the anteroposterior direction, and 2.7 mm in the craniocaudal direction.

The study found lack of correlation between Primovist^®^ MRI‐based delivery accuracy assessment and interfractional motion of three regions considered in this study (liver, FLR, and PTV). The complexity of liver motion which includes interplay effects between heterogeneous deformations and effects of breathing motion is likely the main reason for this lack of correlation. Particularly, interfractional motion alone is not a predictor of liver SBRT delivery accuracy, especially when looking at interfractional motion of the entire liver organ.

An isotropic PTV margin of 5 mm was used for treatment in our study. According to direct evaluations of liver SBRT spatial delivery accuracy, a 5 mm treatment margin would appropriately consider treatment errors, where the median 3D vector magnitude of delivery accuracy was observed to be 4.3 mm. Local control was achieved in 80% of lesions at 14.2 months, thus suggesting a high local control rate for the margins used in the study.

The following limitations are acknowledged. This study was limited by its small sample size. However, this is not uncommon for studies of this nature, as evidenced in Table 2. Although clinically useful for estimation of spatial dose delivery accuracy, Primovist^®^‐enhanced MRI scans are yet to be adopted as a clinical standard for widespread use in liver SBRT treatment regimens. The second limitation is the assessment of liver SBRT delivery accuracy using only the COM shift error. We chose this metric to compare our results against past studies. Furthermore, the automated framework for the hypo‐intensity localization allows for error quantification with respect to COM shifts. Target delineation was not considered since both of our approaches use the same set of contours. Finally, the evaluation of the DIR for the registration of pCT and CBCT was limited to qualitative assessment. The liver region on the CBCT lacks any fiducials or anatomical landmarks; therefore, target registration error could not be quantified within the liver region.

This is the first study to use a novel approach of post‐SBRT Primovist^®^‐enhanced MRI to assess the suitability of treatment margins. The presented study used a PTV treatment margin of a 5 mm isotropic expansion from ITV, which is commonly used in practice.^1^, ^2^, ^7^, ^40^, ^41^, ^42^ Primarily, this study concludes that the treatment margins used in this study are adequate based on Primovist^®^ MRI determined spatial delivery accuracy. The evaluated interfractional motion systematic and random errors were highest in the craniocaudal direction. The directly evaluated liver SBRT delivery error has shown that the anteroposterior direction had the highest COM discrepancy from the intended treatment plan compared with all other anatomical directions. Finally, it was determined that the interfractional motion—particularly when evaluating entire liver region—is not correlated with the liver SBRT delivery accuracy.

## 5 CONCLUSIONS

This study used a combination of the automated segmentation framework and Primovist^®^‐enhanced post‐SBRT MRI for the determination of liver SBRT delivery accuracy and threshold dose associated with the FLR. This is also the first study to assess the impact of interfractional motion on the threshold dose, and the first study to compare interfractional motion trends to that of directly quantified liver SBRT spatial treatment delivery accuracy. The implementation of a post‐SBRT Primovist^®^‐enhanced MRI builds the framework for the evaluation of the appropriateness of current margins in clinical practice. We believe that the framework presented will further encourage a new approach to clinical margin assessment.

## CONFLICT OF INTERESTS

Authors have no conflict of interests to declare.
